# Predicting Insect Migration Density and Speed in the Daytime Convective Boundary Layer

**DOI:** 10.1371/journal.pone.0054202

**Published:** 2013-01-24

**Authors:** James R. Bell, Prabhuraj Aralimarad, Ka-Sing Lim, Jason W. Chapman

**Affiliations:** 1 Department of Agro-Ecology, Rothamsted Research, Harpenden, Hertfordshire, United Kingdom; 2 Department of Entomology, University for Agricultural Sciences, Raichur, Karnataka, India; 3 Environment and Sustainability Institute, University of Exeter, Penryn, Cornwall, United Kingdom; University of Kent, United Kingdom

## Abstract

Insect migration needs to be quantified if spatial and temporal patterns in populations are to be resolved. Yet so little ecology is understood above the flight boundary layer (i.e. >10 m) where in north-west Europe an estimated 3 billion insects km^−1^ month^−1^ comprising pests, beneficial insects and other species that contribute to biodiversity use the atmosphere to migrate. Consequently, we elucidate meteorological mechanisms principally related to wind speed and temperature that drive variation in daytime aerial density and insect displacements speeds with increasing altitude (150–1200 m above ground level). We derived average aerial densities and displacement speeds of 1.7 million insects in the daytime convective atmospheric boundary layer using vertical-looking entomological radars. We first studied patterns of insect aerial densities and displacements speeds over a decade and linked these with average temperatures and wind velocities from a numerical weather prediction model. Generalized linear mixed models showed that average insect densities decline with increasing wind speed and increase with increasing temperatures and that the relationship between displacement speed and density was negative. We then sought to derive how general these patterns were over space using a paired site approach in which the relationship between sites was examined using simple linear regression. Both average speeds and densities were predicted remotely from a site over 100 km away, although insect densities were much noisier due to local ‘spiking’. By late morning and afternoon when insects are migrating in a well-developed convective atmosphere at high altitude, they become much more difficult to predict remotely than during the early morning and at lower altitudes. Overall, our findings suggest that predicting migrating insects at altitude at distances of ≈100 km is promising, but additional radars are needed to parameterise spatial covariance.

## Introduction

Ecologists have argued that universal patterns should emerge from ecosystems despite their apparent complexity [1], [2]. Particularly, patterns should be evident at given spatial and temporal resolutions because individuals within populations either interact individually in some way (parasitism, competition, predation etc) to produce a signal, or the community responds to an exogenous driver such as climate change that cascades through trophic levels [3]–[7]. In this paper we consider mixed populations of numerous insect species migrating at high altitudes (i.e. 150–1200 m above ground level (a.g.l.)) and ask the simple question, can average aerial densities and displacement speeds of 1.7 million insect migrants over the last decade be predicted using reductive linear models?

At and above 100 m a.g.l., the great majority of the daytime fauna in northern Europe comprises Homoptera (primarily aphids), small flies, beetles and parasitic Hymenoptera (parasitoids) [8]. Other groups, such as spiders and mites, can also be found at these heights [9]. Because convection currents circulate small arthropods around in the atmosphere, the fauna is not dissimilar to that captured by suction traps at a much lower height of 12.2 m a.g.l. in which parasitoids, flies and aphids dominate a broad range of invertebrate taxa [10]–[11]. These species are either beneficial to agriculture (e.g. aphid biocontrol agents such as parasitoids, hoverflies, carabids and ladybirds), are pests or disease vectors (most aphids and some mites and flies), or contribute substantially to biodiversity [8], [11].

Periodicity is a feature of flying insects which tend to have quite predictable diel activities, causing fluxes throughout the 24-hour period [12]–[14]. These diel fluxes are largely in response to temperature and light intensity changes, the effects of which are evident during dawn, the middle of the day, and at dusk, when pronounced density peaks are apparent [13]–[17]. In fine weather, insects take advantage of updrafts produced in unstable convective atmospheres and are then transported downwind at speeds often greatly in excess of their self-powered flight speeds [16], [18]–[20]. Day-active species generally descend before dusk, but on some occasions when air-temperatures remain particularly warm at high-altitude daytime species have been known to remain aloft [12], [21]. However, the night migratory flights are usually restricted to a different set of nocturnal insects, and these often become concentrated into layers as a result of the more stable atmosphere in the nocturnal boundary layer which allows insects to rapidly traverse hundreds of kilometres in fast-moving winds [13], [18], [20], [22]–[25]. We do not consider nocturnal movements any further here however, but instead concentrate on the daytime phase which is less well understood.

With such a diverse fauna in the air and with each species or group seemingly conditioned to have discrete flight behaviours that include preferences for height, time and meteorological conditions, it might be expected that general patterns would be elusive. Indeed this is true within the flight boundary layer [FBL] below about 10 m [26]. Above this height, ‘noisy’ density profiles give way to two of the most compelling patterns. Firstly, log density of insects declines close to a linear fashion with log height, generating a negative slope that emerges as a result of changes in atmospheric stability [12], [27]. The slope declines rapidly when the boundary layer is stable, and is markedly shallower if the atmosphere becomes convective [27]. This is an important finding because it indicates that empirical models should be able to relate measures of atmospheric stability, notably temperature and wind speed, to patterns of migration. The log density-log height relationship encapsulates insect populations in the vertical plane and may even be universal above a given height.

In the horizontal plane, Taylor's Power Law is manifest and simply describes log-transformed variance in abundance as a function of log transformed mean abundance [28]. The law was developed from measurements of insect density in the air in collaboration with C.G. Johnson in the 1950s [12]. Their earlier collaboration fuelled later entomological work on mean-variance relationships from aphids caught above the FBL [29]–[31]. In this horizontal plane at heights of 12.2 m, Bell et al. [32] recently described the abundance-occupancy relationship for 170 species of aphids migrating over the United Kingdom, showing that the occupancy and continuity (persistence) of aphids is a function of their log abundance which generates sigmoidal curves with varying lower and upper asymptotes.

In this paper we attempt to model insect densities and their speeds measured by radar at altitudes between 150–1200 m to test a biological hypothesis concerning the period when solar heating of the ground produces rising thermals that generate convective plumes. Our motivation is purely to reveal migration predictability that can be generalised at the community level. Our hypotheses are that exogenous drivers are linearly related to average aerial densities and displacement speeds. We use a 10-year dataset, the longest continuous time series of high-flying diurnal insects in the world, to model 1.7 million radar-detected insect targets that have masses between 5–700 mg. The novelty of this work is that whilst there are hundreds of studies of insects and spiders migrating within their FBL (i.e. usually ∼0.5–10 m; [9], [26], [33]–[34]) there are few empirical non-invasive surveys of day-flying insects within the convective boundary layer [CBL] above the level of the FBL (Ecological: [13], [19], [23]; Meteorological: [35]–[36]). These latter studies either focus on a time-series of just a few days when either particular meteorological, or insect phenomena, were apparent or are purely meteorological. Thus, our study is unique in providing a long time series analysis with mixed meteorological and temporal effects on insect densities and displacement speeds.

## Materials and Methods

### Deriving Data From Vertical-looking Entomological Radar (VLR)

**Figure 1:**
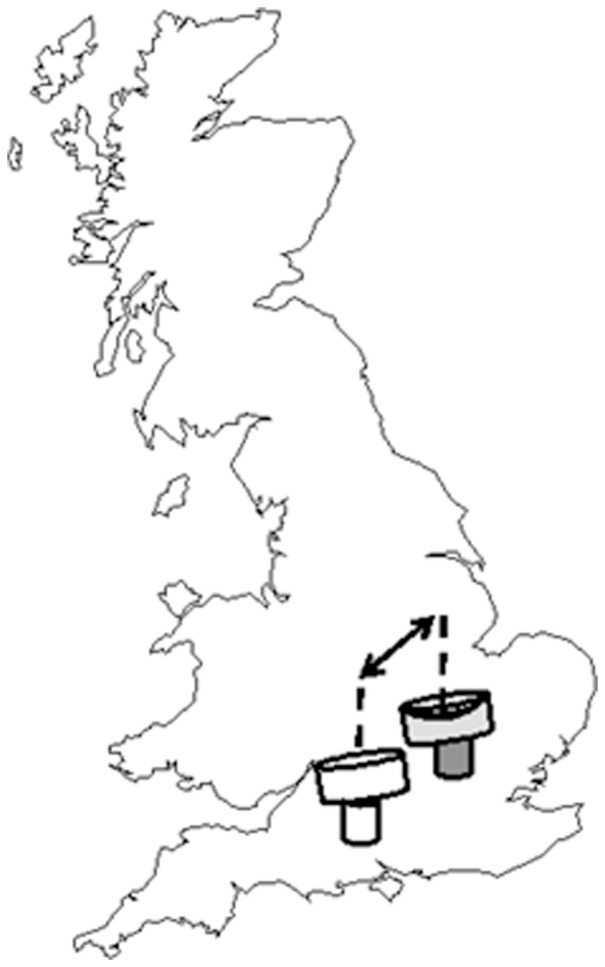
Location of the vertical-looking radars. The radars at Rothamsted, Hertfordshire (shaded) and Chilbolton, Hampshire (white-filled) are shown. The arrow indicates that the Euclidean distance between the sites is 104 km.

Data were derived from daily radar observations at two altitude ranges (150–300 m and 600–1200 m a.g.l.) using Rothamsted Research's vertical-looking radar (VLR) located in Harpenden (51°49′N; 0°22′W; Fig. 1) [15]. Target insects >5 mg migrating through the radar beam are automatically and individually-resolved on a cycle of 5 minutes, once every 15 minutes, 24 h a day using a novel iterative procedure based on components of their complex Fourier transformations [37], [38]. The process yields the horizontal speed, displacement direction, orientation and three radar scattering terms of the target and also calculates the distance of closest approach to the beam's axis of rotation and the time that this point was reached. All these parameters are then used to create a simulated signal and the correlation between the simulation and the actual radar return provides a quantitative estimate of how well our model has described the target [37]. It should be noted that targets are modelled as ‘insects’ as they do not have unique cross-sectional areas that would allow a species identification. It has been well established through aerial netting that these ‘insects’ collectively comprise diurnally-active flies, beetles, and true bugs, while the occasional large insects (>50 mg) are most likely butterflies, dragonflies and grasshoppers [13], [15]–[16]. In the process of the procedure, the radar algorithms also filter out non-biological bodies such as raindrops, radar ‘chaff’ and aerial detritus, as well as bird and bat targets [39]. Ballooning spiders, flying aphids and other micro-insects fall below the minimum mass threshold (≈2 mg) for radar detection and are thus not included in our analyses [8], [15]. Further, we excluded any target that had a mass greater than 700 mg as these are unlikely to be an insect [24]. Target insects were automatically logged and stored in a database ready for extraction.

### VLR Data

**Figure 2:**
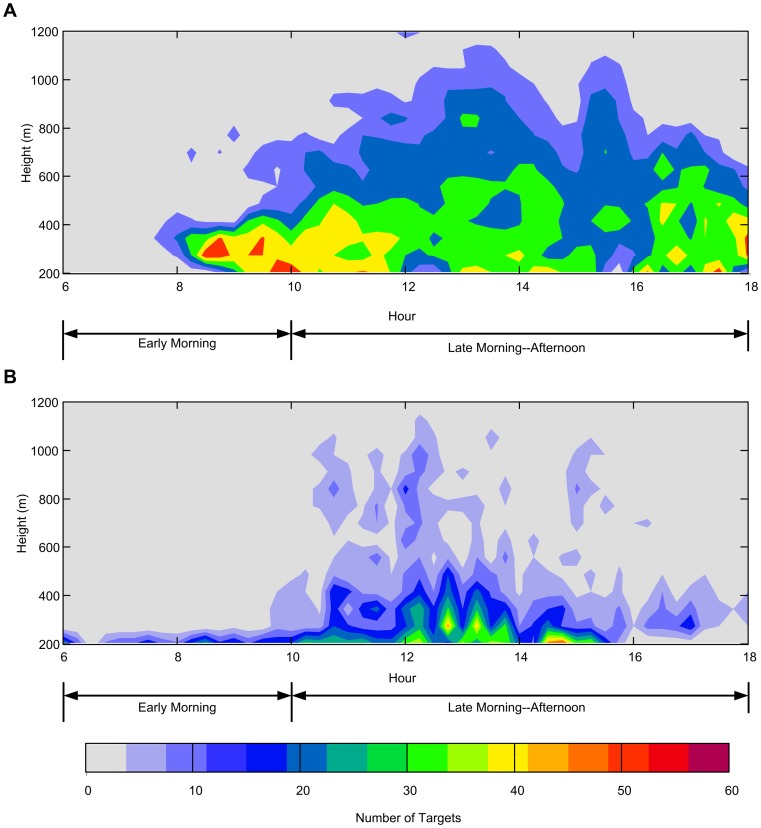
Time/height plots for numbers of insects recorded by a VLR at Cholbolton, Hampshire, UK. The colour scale bar refers to the number of individually-resolvable insects detected by the radar at each sampling height in each 5-minute period. The X-axis shows time of day (GMT), and the ‘early’ and ‘late’ analysis periods are indicated. A. Insect densities on a warm day (05 September 2004), when air temperatures at 10 m, 150 m and 600 m were 24.7°C, 22.8°C and 18.5°C respectively. B. Insect densities on a much cooler day (15 September 2004), when air temperatures at 10 m, 150 m and 600 m were 15.4°C, 13.2°C and 8.8°C respectively. Substantial density was constrained to time-periods and altitudes where air temperatures were relatively warm. Air temperatures were obtained from the UK Met Office's ‘Unified Model’ [14].

We split daily data into ‘early morning’ (06:00–10:00 GMT) and ‘late morning-afternoon’ (10:00–18:00 GMT) to provide a contrast in potential atmospheric conditions between sunrise and a period around midday when the thermal input was likely to be much higher (Fig. 2). Firstly, we sought to ascertain which meteorological variables predict migration intensity and speed at the lower altitude range (150–300 m) during the ‘early morning’. Concomitantly, we asked if the same meteorological predictors were still relevant to insects flying at a much higher altitude (600–1200 m) during the late morning-afternoon period, hereafter denoted as the ‘late period’, when a large number of insects were known to be airborne and at altitude when air temperatures were high (Fig. 2). In both cases, we modelled the total density of insects and their average displacement speed (i.e. their speed in relation to the ground) as separate responses.

A single value for both density and speed was derived for each early and late period (during each day) over five months (May-September) for 10 years (2002 to 2011). All density values are expressed as counts per 10^7^ m^3^ and all displacement speeds as m s^−1^. Aerial densities were corrected for the volume of air sampled, automatically taking account of wind speed. Only days with >10 radar-detected insects in the relevant altitude and time range were included in the analyses. Associated with early and late periods were a set of temporal factors: each period within each radar day was associated with a given week number, a month and year. It is important to note that there are normally few insects at high altitude in the early morning period but instead there is a progression of insects, known as a discharge, from the lower altitudes to the upper altitudes as convection develops toward midday. These high flyers then usually ‘fall out’ as nightfall approaches, most landing before sunset [12]. Hence, our experimental structure cannot be a fully crossed design, but instead it is fractional and reflects the biological nature of migration in the CBL.

The Rothamsted VLR was then compared with our other VLR, situated at the Chilbolton Facility for Atmospheric and Radio Research, Hampshire (2004 onwards) (51°09′N; 1°26′W; Fig. 1) in an attempt to derive parameters on the predictability of insect densities and displacement speeds in southern Britain. The radars are assumed to be independent because insect migrants are highly unlikely to travel between the sites due to large-scale circulation patterns. Data from Chilbolton was cross-tabulated to meet data requirements described above for Rothamsted (i.e. altitude, mass, period).

### Meteorological Data

Air temperature and wind speeds at relevant altitudes were estimated using the operational mesoscale version of the UK Met Office's numerical weather prediction model, named the ‘Unified Model’ (hereafter referred to as ‘MetUM’; see http://www.metoffice.gov.uk/research/modelling-systems/unified-model). For our analyses, we used MetUM mean outputs from 190 m and 770 m for the ‘early morning’ and ‘late morning-afternoon’ periods respectively.

### Statistical Analysis

#### Data exploration

Given that high-altitude daytime insect densities and their associated displacement speeds are little understood, we use some basic models to examine their structure. Lorenz curves and associated statistics (i.e. Gini and asymmetry co-efficients) were used to examine the change in inequalities in each response [40]. Briefly, if all densities and velocities were the same the Gini coefficient describing the size of area of inequality from the Lorenz curve would be zero – that is, the relative mean difference would be the same. Additionally, bias can be examined using the axis of symmetry which has the coordinates (1, 0) to (0, 1). If S>1, the point where the Lorenz curve is parallel with the line of equality will also be above the axis of symmetry. Correspondingly, if S<1, the point where the Lorenz curve is parallel to the line of equality is thus below the axis of symmetry. For both Gini and asymmetric co-efficients, 95% bootstrap confidence intervals were produced. Pearson moment correlations were used to express changes in density as a function of increasing displacement speeds.

#### Regression models with meteorological variables

The total density of insects in the air and displacement speeds of those insects once airborne were studied using generalized linear mixed effect models (GLMMs). GLMM used the method by Schall [41] in which models were fitted using penalised quasi-likelihood [42]. Early period densities; late period densities; early period displacement speeds and late period displacement speeds were the four responses that were considered. The fixed effects were temperature and wind, at the relevant heights to the response. The dispersion parameter (Ø) was estimated from the residual mean square which should approximate unity. We sought to minimize the difference from unity by removing redundant random effects. The maximal random effects structure was the product of all the effects (i.e. year*month*week) which allows for there to be exceptional weeks or months as separate interacting terms as well as individual single effects. It was quickly established, however, that over the decade of the time series that month was a redundant effect in the models which inflated Ø. Year.week was used as a single random effect and gave the closest approximation to a dispersion parameter (Ø) of unity for all models: this model infers that only the combined effect of year.week has a profound effect on the models, and, as additive terms year and week are not needed.

Within GLMMs *log(μ_ijk_*) is the expected response (on the link scale: log or identity), that insects are subject to prevailing weather, where *i* is the unit for *j* response (displacement speed or density) in *k* period (early, late and in which the height of the radar is synonymous with period and thus not crossed) and temperature (*l* = −1,0,1,…27) and wind (*m* = 0,1,2,…21) are the fixed effects of interest. The random effect *b* included only the interaction between year (*x* = 1,2,3,…10) and week (*z* = 1,2,3…21)) and estimated the variance component. Thus, the general form of the maximal model was:
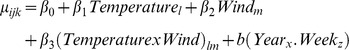



Distributional assumptions differed between total density of insects and their average velocity. Due to the very large variance heterogeneities and overdispersion in the errors, total density fluxes were modelled using the negative binomial with an aggregation parameter of unity and a logarithmic link [43]. Velocity measurements were instead modelled with a normal distribution and an identity link. We used reverse model selection procedures in our search for the parsimonious model. The Wald statistic assessed the contributions of individual terms in the fixed model: if a term produced non-significant values of P>0.05, the variable(s) was subsequently removed from the model, and the model updated until all terms had significance values P<0.05. Velocity measurements did not have very large variance heterogeneities but were instead modelled with a normal distribution and an identity link, producing very similar parameters to that of a linear mixed-effects model (not shown).

Throughout the text, subscripts *e* and *l* refer to the ‘early period’ and ‘late period’ respectively. These two subscripts are then combined with the notation for displacement speeds (*V*) and density (*D*) responses in the regression models or to parameters (i.e *G*, *S, r* and mean) in data exploration section.

#### Regression models between sites

Discrete regression models for both densities and speeds were developed for Chilbolton using ordinary least-squared regression models (OLS). The Rothamsted VLR was the response and Chilbolton was the explanatory variable. In these models the sole aim was to establish how predictable VLR data were from a separation distance of 104 km away. We consider insects directly and do not consider analysing the residuals of the model to look at insects having taken account of the prevailing weather. The body of evidence suggests that the convective plumes that the insects are using to remain aloft are scaled at much finer levels (<5 km) than the distance between the radars ([19], and see discussion later). Speeds were log transformed and densities square-root transformed prior to analyses.

## Results

### Data Exploration

**Figure 3:**
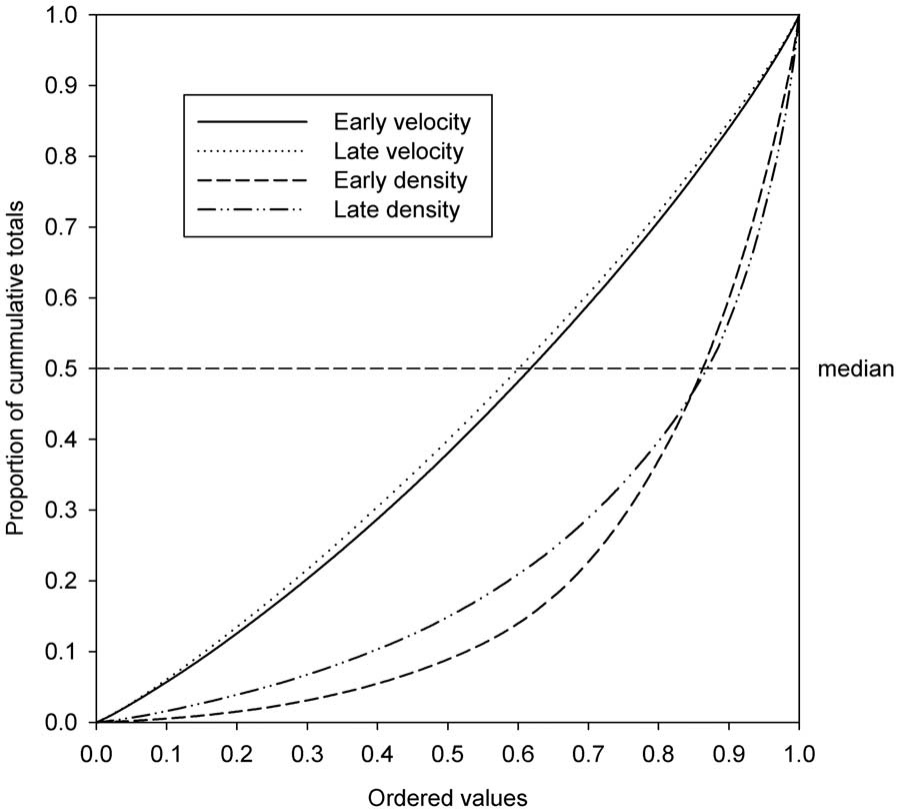
Lorenz curves for insect displacement speeds and densities by period.

Insect displacement speeds tended to be close to symmetrical (*S*_e_ = 0.9540; 95% CI: 0.916, 0.997; *S*_l_ = 0.9797; 95% CI: 0.923, 1.050) and near to the line of equality (*G*_e_ = 0.1679; 95% CI: 0.159, 0.175; *G*_l_ = 0.1446; 95% CI: 0.134, 0.154). All the indications are that the distribution of displacement speeds, irrespective of time of day or flight altitude, was not skewed but uniform because both the relative mean difference between all values was broadly even and the distribution was closely aligned to the axis of symmetry (Fig. 3). As might be expected, at a higher altitude and during a later period of the day, insects travelled faster on average (mean = 14.29 m s^−1^, se ±0.18) than those individuals earlier in the day and at lower altitude (mean = 7.83 m s^−1^, se ±0.08).

Insect densities tended to depart radically from the line of equality (*G*_e_ = 0.6037; 95% CI: 0.588, 0.625; *G*_l_ = 0.5456; 95% CI: 0.510, 0.582), indicating that distributions were dominated by comparatively few but large values. Early morning densities were particularly asymmetrical about their distribution (*S*_e_ = 0.8786; 95% CI: 0.844, 0.919), although this asymmetry dissipated out by the later period (*S*_l_ = 1.049; 95% CI: 0.989, 1.094). Approximately, 50% of the insect density values above the median contained only ≈13% of the overall insect densities for both early and late periods (Fig. 3), suggesting that there are occasions when insect densities are super abundant but such events are uncommon. The mean densities in the early morning are a particularly extreme case (mean = 2219, se ±99.69 per 10^7^ m^3^) in which the range is vast (min = 15.37; max = 16,409 per 10^7^ m^3^) although values remain modest even by the 75% percentile (Q_2_ = 3146 per 10^7^ m^3^). Later on in the day, the range of insect densities is relatively small in comparison to the early period (min = 9.74; max = 1064 per 10^7^ m^3^).

The relationship between displacement speeds and densities was negative: as speeds increase towards the maximum for the period (early max = 17.78 m s^−1^; late max = 27.48 m s^−1^), insect densities decline. This decline is twice as fast in the morning than in the afternoon (*r_e_* = −0.2562 P = <0.001; *r_l_* = −0.1444; P = 0.002).

### Regression models

The evidence for an interaction between temperature and wind that would then influence insect displacement speed or migration intensity was absent for both periods (temp.wind P>0.05: V_e_ [F = 0.52, ddf = 709.0, P = 0.470]; V_l_ [F = 1.78, ddf = 328.6, P = 0.183]; D_e_ [F = 2.15, ddf = 756.4, P = 0.143]; D_l_ [F = 0.31, ddf = 439, P = 0.575]). Instead, for insect displacement speeds it was possible to generate a very simple model that described how wind increased displacement speeds for early and late periods respectively (V_e_[F = 1290.36, ddf = 736.2, P<0.001]; V_l_[F = 593.48, ddf = 335.0, P<0.001]). In this model there is no requirement to include a temperature component (V_e_ [F = 0.68, ddf = 750.2; P = 0.411], V_l_ [F = 1.22, ddf = 384.4, P = 0.271]).

Both wind (D_e_ [F = 263.36; ddf = 742.5; P = <0.001]; D_l_ [F = 17.31; ddf = 428.0; P = <0.001]) and temperature (D_e_ [F = 240.19; ddf = 740.7; P = <0.001]; D_l_ [F = 41.96; ddf = 433.9; P = <0.001]) have a highly significant effect on insect densities which fall with increasing wind (early: −0.18 ±se 0.01; late: −0.05 ±se 0.01) and rise with increasing temperatures (early: 0.212 ±se<0.01; late: 0.09 ±se 0.01). Notably, the rate of change is faster during the early period compared to the late for both fixed effects.

### Predicting Rothamsted VLR dynamics remotely

**Figure 4:**
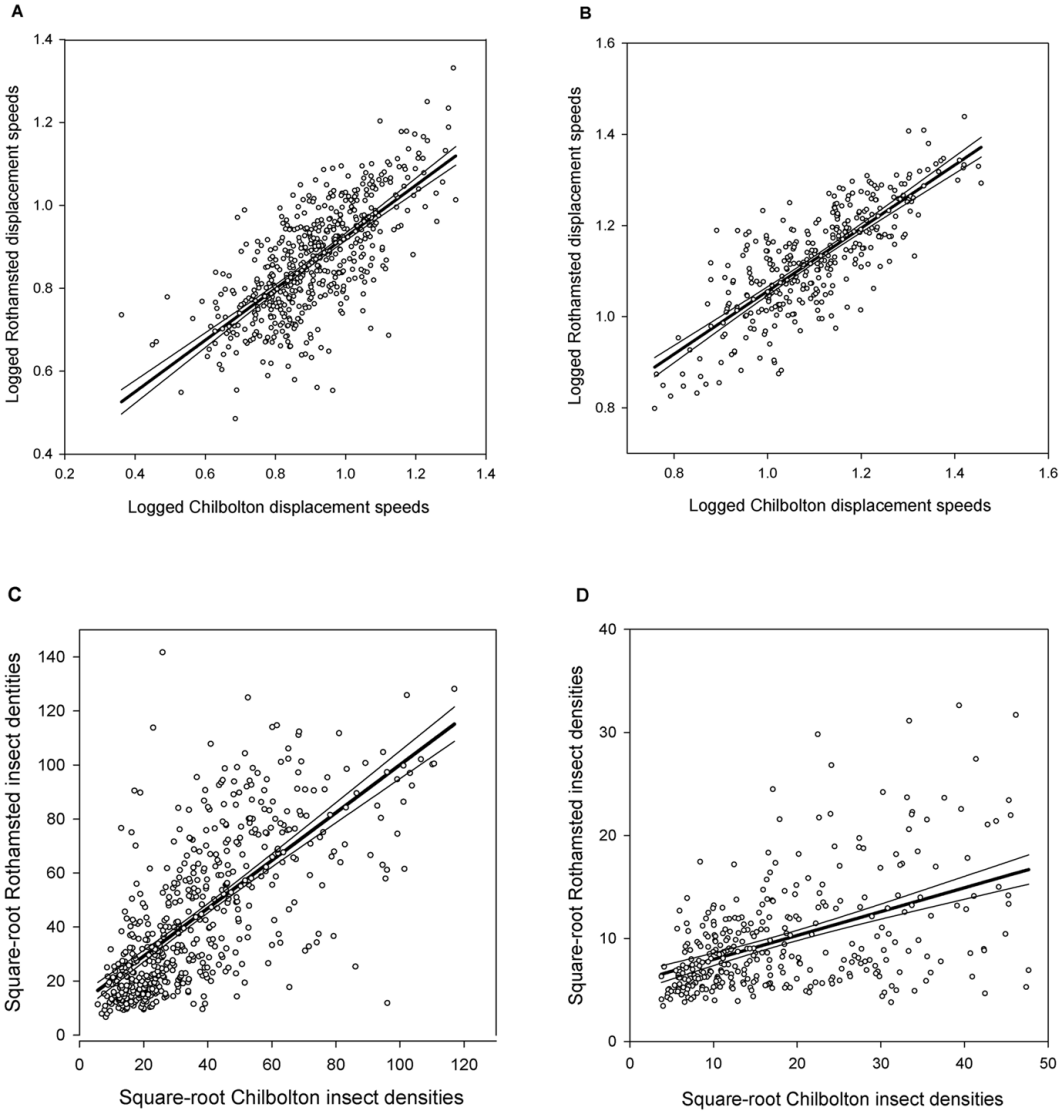
Linear regressions. Fitted and observed relationships with 95% confidence intervals between Chilbolton and Rothamsted logged displacement speeds by period: A. early period; B. late period, and, Chilbolton and Rothamsted square-root transformed insect densities by period: C. early period, D. late period.

For all regression models, Chilbolton was highly significant at predicting the dynamics of the prevailing insect displacement speeds (V_e_ [F_1,550_ = 557.15; P<0.01; *r* ^2^ = 0.502]; V_l_ [F_1,340_ = 564.19; P<0.01; *r* ^2^ = 0.623]) and densities (D_e_ [F_1,550_ = 513.87; P<0.01; *r* ^2^ = 0.48.2]; D_l_ [F_1,341_ = 101.05; P<0.01; *r* ^2^ = 0.226]) at Rothamsted, 104 km away. Insect displacement speeds have very similar slopes throughout the day (V_e_ β = 0.623±0.026; V_l_ β = 0.692±0.029) (Fig. 4A, 4B), but densities differ markedly (D_e_ β = 0.886±0.039; D_l_ β = 0.231±0.023). Further, as evidenced from the models, when insects are migrating in a convective atmosphere that is well developed during the late morning and afternoon, the migrating population becomes more difficult to predict even though the numbers are considerably smaller (Fig. 4C, 4D).

## Discussion

To date the relationship between migrating aerial insects across space is little understood, mostly due to the technicalities of concurrently measuring the aerial biomass without significant bias. We found that ≈50% of the variance in radar-detected insect densities can be explained by a predictor radar ≈100 km away (Figs. 1 and 4A, 4B, 4C, 4D) which bodes well for models that aim to produce forecasts of flying insects. The size of the variance captured was hypothesized as being a function of the difference in landscape between these sites, particularly the surface characteristics (water, soil, vegetation and the built environment). For example, there is substantially more development around Rothamsted which sits on the boundary between urban and rural environments, compared to Chilbolton which is distinctly more rural. These surface characteristics are well known to determine the scale at which individual convection plumes operate [44]–[46]. In the horizontal plane, the size of the plume is known to be scaled to quite a fine resolution, much less than the Euclidean distance between the sites we studied (i.e. 105 km). For example, Kitchen and Caughey [47] suggest that the scaling lies somewhere between 100 m to 1 km although Bénard-type convective cells are 3–4 km across [48, Don Reynolds *pers. comm*.] which is not dissimilar to the size of more general plumes reported by Geerts and Miao [19]. From a bottom-up perspective, landscape heterogeneities that have an effect on the atmosphere tend to be scaled at distances greater than this, perhaps up to 5 km [46], [49].

Thus, the scale at which landscape surface varies will directly impact the convection process and our models reflect that implicitly. Deforestation or wide-scale agricultural management over large areas for example, may expose the soil surface to greater thermal input and stronger convection as a result [44], [50], which may promote migration. Topography also plays an important role: towards the west coast and on upland sites in the UK, surface winds are characterised by their high speeds [51] which may depress the onset of migration in these regions. Variation in both topography and surface characteristics determine what happens in the vertical plane too. Here, thermal plumes rise in the CBL in response to increasing surface temperatures and these convective updrafts carry warm air which cools, slowing in the rate of ascent as it rises to heights of up to ≈1–2 km until it finally loses its identity [48].

Given all these convective complexities, our finding that ≈50% of the variance in radar-detected insect densities can be explained by a predictor radar ≈100 km away (Figs. 1 and 4A, 4B, 4C, 4D), is not only somewhat surprising, but it also highlights a need for more spatially replicated data to establish scaling rules and determine levels of spatial synchrony. We acknowledge that this is beyond the limits of our study but what we can conclude is that the best predictors for an entomological radar will be a radar that is sited in a landscape with the same configurational and compositional components [52]. Any prediction of the aerial biomass over space and time is, at the very least, a three-way interaction between the convective landscape, habitat and differences in individual behaviour (e.g. phenology/circadian rhythms). This is perhaps why we are better able to estimate speeds of the animals in transit than total densities because speed can be reduced to pure physics whereas densities cannot. Whilst diurnal insect displacement speeds we recorded agreed well with previous studies using radar [13], they correlate strongly with the actual speed of the wind at altitude [35]–[36]. For example, the mean displacement speed of the carabid beetle *Notiophilus biguttatus* between150 to 408 m was 7.3 m s^−1^, and the mean wind speed was 4.8 m s^−1^ [53], indicating that the mean air speed of the migrating *N. biguttatus* was small and around 2.5 m s^−1^.

Speed and density were shown to be negatively related. Weak-flying, small-bodied (<10 mm) insects not only decline with increasing height [27] but we show that densities fall with increasing wind speed and rise with increasing temperatures (Fig. 2). The rate of change is fastest in the morning when the atmospheric stability is in a non-equilibrium state [48] yielding occasions, albeit rarely, when insect densities are super abundant at altitudes between 150–300 m (i.e. ≈16,000 per 10^7^ m^3^ period^−1^). Considering all early mornings studied, the majority yield only modest densities, much less than 3,500 insects (75% percentile) which is a product of non-ideal take-off conditions and concomitantly weaker thermals in a windier atmosphere.

The interplay between speed and density is an interesting facet that may have arisen from turbulence which redistributes the fauna laterally. Alternatively, the fall in numbers as speeds increase may simply indicate that for an increasing proportion of the potential aerial fauna, conditions are simply not suitable for flight which is either not initiated or curtailed [12]. Flight conditions, particularly temperature-insect relationships at altitude, have been described formally since Johnson [12]. More recently Wood et al. [23] established that warmer days are associated with more aerial migration and that the minimum threshold was around 13–14°C. Hotter days are associated with more frequent, longer-lasting and deep convective plumes allowing more insects to be spread through the CBL (Fig. 2). Interestingly, our results also show that at a higher altitude and during a later period of the day, insects travelled horizontally twice as fast on average (14.29 m s^−1^, se ±0.18) than those individuals earlier in the day and at lower altitude (7.83 m s^−1^, se ±0.08), which is a function of wind speed and consistent with transport by updrafts (i.e. thermals that are driven by convection) within the CBL [23].

Revisiting our original hypotheses regarding the prediction of average aerial densities and displacement speeds and also in light of the complex meteorology discussed so far, we found that insect densities become much more difficult to predict remotely, especially by the afternoon. This is most likely because during fine weather in summer, morning may often begin with the erosion of any stable nocturnal temperature inversion and by 11am convection may increase to heights of 750–900 m in the UK depending on location, wind and solar input [54]. In response to these thermals, there may be large numbers of invertebrates that have taken advantage of the updrafts and have risen to ≈800 m by late morning [13] —the weather now becomes increasingly heterogeneous and varying in spatial extent. Within the plumes, wind speeds are driven by convection wherein 80% of the turbulence is explained by thermals with updrafts reaching 1–2 m s^−1^ in the mixed layer below the cloud base, but averaging 0.5 m s^−1^ [36], [47], [55]. Above the base of the cloud when convection has reached its highest extent by mid-afternoon, the atmospheric boundary layer may reach heights of 1–2 km in which wind speeds approach 5 m s^−1^ at the top of the clouds [23], [36], [47]. But by afternoon, the way in which the numbers of insects are then redistributed in these plumes becomes quite difficult to predict remotely at scales greater than the plumes themselves (i.e. ≈5 km). Thus it is clear that entomologically the dynamics are not consistent across space and that significant spatial covariance may be ultimately elusive at regional scales.

Finally, it seems a widely held misconception that the act of migrating is a risky redistribution strategy, given the heights individuals achieve. As Johnson [12] states, the cost associated with moving large distances is low at altitude because most insects are vagile even well above 1000 m where the atmosphere is quite cold. The real cost of migration is deferred because whilst the actual cost of transit is low, the great majority of insects may experience the cost of moving during and after deposition [56] thus advocating a mixed Evolutionary Stable Strategy for long distance movements. For example, Ward et al. [57] showed that after migrating, less than 1% of the bird cherry-oat aphid populations were able to find hosts. However, larger insect migrants are known to utilise wind currents in a highly efficient manner, thus reducing the probability of being dispersed to unsuitable habitats [20], [24], [58].

## Conclusions

Insect migration studies have made major contributions to ecology [3], [12], [28]–[32] and have been complemented by more general studies on effects of surface landscape heterogeneity at various scales [50] and the meteorology itself [35]–[36]. Migration studies are important because of the sheer abundance of insects that use the CBL to move. For example, Chapman et al. [15] established that for the southern U.K. the size of the migrating populations is equivalent to 3 billion insects km^−1^ month^−1^ comprising pests, beneficial insects and other species that contribute to biodiversity. Indeed it is because of the very nature of migrating pests and beneficial insects that it is prescient that we understand their dynamics to better deliver food security and future pest control. We have shown that it is possible to generalize about the aerial fauna over a long time series which is a significant step forward in the ecology of aerial migrants. Meteorological mechanisms, principally related to wind speed and temperature, drive variation in density and displacements speeds with increasing altitude. A strong linear relationship was apparent between sites separated by ≈100 km, 10-fold larger than previously expected (i.e. 1–5 km), which will go some way in meeting the challenge of forecasting migration.
